# Plant Molecular Farming: A Viable Platform for Recombinant Biopharmaceutical Production

**DOI:** 10.3390/plants9070842

**Published:** 2020-07-04

**Authors:** Balamurugan Shanmugaraj, Christine Joy I. Bulaon, Waranyoo Phoolcharoen

**Affiliations:** 1 Research Unit for Plant-Produced Pharmaceuticals, Chulalongkorn University, Bangkok 10330, Thailand; Balamurugan.S@chula.ac.th; 2 Department of Pharmacognosy and Pharmaceutical Botany, Faculty of Pharmaceutical Sciences Chulalongkorn University, Bangkok 10330, Thailand; 6176101033@student.chula.ac.th

**Keywords:** biopharmaceuticals, molecular farming, *Nicotiana*, plant production system, plant-derived protein, recombinant protein, transient expression

## Abstract

The demand for recombinant proteins in terms of quality, quantity, and diversity is increasing steadily, which is attracting global attention for the development of new recombinant protein production technologies and the engineering of conventional established expression systems based on bacteria or mammalian cell cultures. Since the advancements of plant genetic engineering in the 1980s, plants have been used for the production of economically valuable, biologically active non-native proteins or biopharmaceuticals, the concept termed as plant molecular farming (PMF). PMF is considered as a cost-effective technology that has grown and advanced tremendously over the past two decades. The development and improvement of the transient expression system has significantly reduced the protein production timeline and greatly improved the protein yield in plants. The major factors that drive the plant-based platform towards potential competitors for the conventional expression system are cost-effectiveness, scalability, flexibility, versatility, and robustness of the system. Many biopharmaceuticals including recombinant vaccine antigens, monoclonal antibodies, and other commercially viable proteins are produced in plants, some of which are in the pre-clinical and clinical pipeline. In this review, we consider the importance of a plant- based production system for recombinant protein production, and its potential to produce biopharmaceuticals is discussed.

## 1. Introduction

Recombinant proteins are complex exogenous (“foreign”) proteins that are produced in expression hosts, and mainly used as medical diagnostic reagents and in human healthcare as vaccines, drugs, or monoclonal antibodies [1]. The prominent role and increasing market demand for high-value recombinant proteins in novel drug discovery creates an opportunity for the development of various protein expression hosts to manufacture proteins by following the existing rigid standards laid down for veterinary and human applications. The industry is focusing mainly on already established production platforms using prokaryotic and eukaryotic expression host systems such as *Escherichia coli*, a selection of yeast, insect, and mammalian cell cultures, due to their well-defined processes in-line with current good manufacturing practice (cGMP) [2]. Moreover, industrially established mammalian and other cell cultures have stringent regulatory approval in place, which hinders the industrial acceptance of the new technology or production system. Bacterial expression systems offer rapid production with high product yield, whereas *Saccharomyces cerevisiae* and *Pichia pastoris* (yeast) offer post-translational modifications (PTMs) which are essential for functional activity of the recombinant proteins [3]. The majority of the approved recombinant biopharmaceuticals are produced in mammalian cell lines [4]. However, all the production systems have their own merits and setbacks such as production time, high operating costs, protein yield, chances of contamination with pathogenic microorganisms, limited post-translational modifications, and regulatory approval. In order to compete with the established platform, the new expression system must have unique advantages that can overcome the limitations of the existing ones. Plants offer several potential benefits over conventional expression platforms and prove the reliability of the system for the production of highly valuable proteins. Advancements in plant molecular farming approaches in the recent decade have made plants an attractive manufacturing system that can even achieve commercially relevant production levels in a short period [1,5,6]. As the progress is continuously being made in this ever-growing field, here in this review, we summarize the importance and prospects of plant expression systems for the cost-effective production of recombinant proteins. Potential vaccine candidates, monoclonal antibodies, and industrial enzymes expressed in plants are also described.

## 2. Plant Expression Platform

Plants were utilized for the expression of recombinant proteins from the late 1980s [7]. Since then, the plant expression platform has faced several hurdles, until recently the first plant-based product “Elelyso” was commercialized by Protalix Biotherapeutics for the treatment of Gaucher’s disease in 2012 [8]. The practice of using plants for high-value recombinant protein production ranging from pharmaceutical therapeutics to non-pharmaceutical products such as antibodies, vaccine antigens, enzymes, growth factors, research or diagnostic reagents, and cosmetic ingredients [9] has improved over time and advanced significantly in recent decades, which in turn has led to a major paradigm shift in the pharmaceutical sector. The technology has rapidly developed, and the potential drawbacks associated with plant molecular farming during their early stages of development, including the need for a high protein expression level and efficient downstream processing, have now been achieved. The advantages of plant expression platforms are cited in several earlier reports showing head-to-head comparisons with other existing platforms (Table 1) [10,11,12,13,14,15,16,17,18]. The key advantages of all plant-based systems are easy cultivation, low expenses, low or no pathogen load, rapid mass production of recombinant proteins, and the ability of the plants to assemble complex proteins with eukaryotic-like post-translational modifications (PTMs) [19]. 

Protein folding is highly essential to retain the biological activity of the recombinant therapeutic proteins. Due to the lack of the protein processing complex and limited capacity for PTMs, proper protein folding cannot be achieved in the prokaryotic expression system [20]. Plants have the capacity to assemble and perform PTMs of large multimeric proteins required for their functional biological activity. However, plants lack the authentic human *N*-glycosylation processing mechanism which has been overcome by the glycoengineering approaches towards the synthesis of targeted human and non-human structures to increase product homogeneity, quality, and quantity [21,22].

The *Nicotiana* genus is often used for genetic transformation studies due to its growth rate and easy genetic manipulation. Most of the recombinant proteins such as pharmaceuticals, vaccines, hormones, cytokines, growth regulators, and industrial products are produced in tobacco, which is considered as a molecular biology workhouse of the plant world. *Nicotiana benthamiana* and *N. tabacum* are two common species used for the stable and transient expression of recombinant proteins. Further, several cereal crops, fruits, and vegetables such as rice, maize, lettuce, tomato, potato, and alfalfa were also evaluated for their applicability in plant molecular farming (PMF) depending on the protein target and application [1]. Many plant-produced therapeutic proteins are in pre-clinical and clinical trials and are close to commercialization [23,24]. The strength and bottlenecks of the commercial potential of the plant expression system was critically reviewed and summarized by Schillberg et al. (2019) [2].

**Table 1:** Available expression platforms for recombinant protein production with potential advantages and disadvantages (adapted from Shanmugaraj et al., 2020) [25].

Expression System	Advantages	Disadvantages
Bacteria	Easy to manipulate Low cost High expression Ease of scale up Short turnaround time Established regulatory procedures and approval	Improper folding Lack of post-translational modifications which may affect the protein function Endotoxin accumulation
Mammalian Cells	Proper folding and authentic post-translational modifications Existing regulatory approval	High production cost Expensive media and culture condition requirements
Yeast	Rapid growth and scalable Easy to manipulate Simple and inexpensive media requirements and culture conditions Post-translational modifications of recombinant proteins	Difficulty in cell disruption due to the thick and hard cell walls Hyperglycosylation of proteins Limited glycosylation capacity
Insect cells	High expression levels Ability to produce complex proteins including secreted, membrane, and intracellular proteins Proper folding and post-translational modifications	High cost and time consuming Expensive media and culture condition requirements
Plant	Rapid and affordable Optimized growth conditions Free from pathogen and bacterial toxin contaminants Economical Post-translational modification somewhat similar like mammalian system	Regulatory compliance Limited glycosylation capacity

## 3. Plant-Derived Recombinant Proteins

The concept of using plants for the production of foreign proteins including pharmaceutical and non-pharmaceutical proteins has been well explored and documented. Many reports proved the ability of in vivo and in vitro plant systems to produce vaccine candidates both for veterinary and human applications and showed that plant-produced antigens elicit potential immune responses in animal models and even confer protection in animal challenge experiments. Examples of the variety of pharmaceutical and non-pharmaceutical proteins expressed in plant systems are illustrated in Table 2 and Table 3.

Tobacco has been engineered to express a variety of antigens in the nucleus and chloroplast including, but not limited to, chikungunya, dengue, Ebola, influenza, and Zika. The transformation protocols for recombinant protein production are also established for fruits and vegetables such as tomatoes and potatoes. Transgenic potatoes expressing the S1 glycoprotein of the infectious bronchitis virus confers protection to chickens upon virus challenge [26]. Leafy crops such as lettuce, alfalfa, and clover have been investigated for molecular farming to obtain the oral delivery of vaccine antigens eliminating purification and injections. The lettuce chloroplast-derived booster vaccine using lyophilized plant cells expressing the poliovirus capsid protein induced neutralization antibodies in mice primed with inactivated poliovirus vaccine (IPV) and conferred protection against all polio serotypes [27]. Plant systems have also been evaluated for the expression of virus-like particles (VLP) of many viruses including norovirus, poliovirus, foot-and-mouth disease virus, influenza, [28,29,30,31], and the potential for plant-derived VLPs to be used as candidate vaccines and reagents has been reviewed in detail elsewhere [18,32,33]. Apart from expressing antigens for human diseases, several antigens for veterinary applications and non-pharmaceutical proteins have also been well tested for expression in plants, and are particularly gaining attention due to the fact that these products can quickly reach the market due to lower regulatory burden [9]. This was clearly evidenced by the commercialization of avidin [34], *β*-glucuronidase [35], and trypsin [36] by the US-based biotechnology company ProdiGene, Inc. The vaccine against Newcastle disease virus (NDV) was the first plant-based poultry vaccine (Dow Agrosciences) that obtained regulatory approval from the United States Department of Agriculture in 2006, opening a new avenue for the commercialization of plant-derived vaccines. Currently, many plant-derived non-pharmaceutical and pharmaceutical proteins are in clinical development. 

Although proof-of-concept and efficacy of many vaccine candidates proved the feasibility and scalability of the robust plant system, it is high time to compete with the established expression systems. Now the plant-based good manufacturing practices (GMP) complaint production facilities such as Fraunhofer (Germany), Kentucky BioProcessing (USA), Medicago (Canada), and Protalix Biotherapeutics (Israel) are available to manufacture GMP materials for human clinical trials. Fraunhofer IME received a GMP license for the production of neutralizing anti-HIV antibody 2G12 in tobacco for phase I clinical testing [37]. The plant molecular farming research community continuously thrives to set up a regulatory framework for plant-derived products. 

**Table 2:** Selected list of vaccine candidates and antibodies expressed in plants against various diseases.

Vaccine Candidates					
Recombinant Protein	Pathogen/Disease	Expression System	Transformation Method	Expression Level	Reference
Hepatitis B surface antigen	Hepatitis B virus	Tobacco (*Nicotiana tabacum*)	*Agrobacterium* mediated (Stable expression/Nucleus)	66 ng/mg of soluble protein	[38]
Structural protein VP60	Rabbit hemorrhagic disease virus (RHDV)	Potato (*Solanum tuberosum*)	*Agrobacterium* mediated (Stable expression/Nucleus)	0.3% of total soluble protein	[39]
Spike (S) protein of transmissible gastroenteritis virus	Transmissible gastroenteritis virus (TGEV)	Tobacco (*Nicotiana tabacum*)	*Agrobacterium* mediated (Stable expression/Nucleus)	0.1–0.2% of total soluble protein	[40]
Hemagglutinin protein of rinderpest virus	Rinderpest virus (RPV)	Peanut (*Arachis hypogea* L.)	*Agrobacterium* mediated (Stable expression/Nucleus)	0.2–1.3% of total soluble protein	[41]
Glycoprotein D (gD) of bovine herpes virus	Bovine herpes virus	Tobacco (*Nicotiana benthamiana*)	Mechanical inoculation (Stable expression/Nucleus)	20 μg/g fresh weight (FW)	[42]
L1 major capsid protein	Human papillomavirus	Tobacco (*Nicotiana tabacum*)	*Agrobacterium* mediated (Stable expression/Nucleus)	2–4 µg/kg FW	[43]
Spike (S) protein of transmissible gastroenteritis virus	Transmissible gastroenteritis virus (TGEV)	Corn (*Zea mays*)	*Agrobacterium* mediated (Stable expression/Nucleus)	13 mg/kg FW	[44]
Spike (S) protein of infectious bronchitis virus	Infectious bronchitis virus (IBV)	Potato *(Solanum tuberosum)*	*Agrobacterium* mediated (Stable expression/Nucleus)	2.39–2.53 µg/g FW	[26]
Anthrax protective antigen (PA)	Anthrax	Tobacco (*Nicotiana tabacum*)	Biolistic method (Stable expression/Chloroplast)	14.2% of total soluble protein	[45]
Hepatitis B virus surface antigen	Hepatitis B virus (HBV)	Potato (*Solanum tuberosum*)	*Agrobacterium* mediated (Stable expression/Nucleus)	8.5 μg/g FW	[46]
Fusion (F) protein of Newcastle disease virus	Newcastle disease virus (NDV)	Corn (*Zea mays* L.)	Biolistic method (Stable expression/Chloroplast)	3.0% of total soluble protein	[47]
F4 fimbrial adhesin FaeG	Enterotoxigenic *E. coli*	Alfalfa (*Medicago sativa* L.)	*Agrobacterium* mediated (Stable expression/Chloroplast)	1.0% of total soluble protein	[48]
L1 capsid protein gene	Cottontail rabbit papillomavirus	Tobacco (*Nicotiana tabacum*)	*Agrobacterium* mediated (Stable expression/Nucleus)	0.4–1 mg/kg of total leaf mass	[49]
Structural protein VP2	Infectious bursal disease virus (IBDV)	Rice	*Agrobacterium* mediated (Stable expression/Nucleus)	40.21 µg/g FW	[50]
Hepatitis B virus surface antigen	Hepatitis B virus (HBV)	Tobacco (*Nicotiana benthamiana*)	*Agrobacterium* mediated (Transient expression)	295 µg/g FW	[51]
Haemagglutinin (HA)	H5N1 (avian influenza virus) & H1N1 (human) influenza strains	Tobacco (*Nicotiana benthamiana*)	*Agrobacterium* mediated (Transient expression)	50 mg/kg FW	[52]
Heat-labile toxin B subunit (LTB)	Enterotoxigenic *E. coli*	Carrot (*Daucus carota*)	*Agrobacterium* mediated (Stable expression/Nucleus)	0.3% of total soluble protein	[53]
Norwalk virus capsid protein	Norwalk virus	Tobacco (*Nicotiana benthamiana*)	*Agrobacterium* mediated (Transient expression)	0.8 mg/g FW	[54]
Structural protein VP1	Foot-and-mouth disease virus (FMDV)	Legume (*Stylosanthes guianensis*)	*Agrobacterium* mediated (Stable expression/Nucleus)	0.1–0.5% of total soluble protein	[55]
HIV-1 Pr55gag Polyprotein	Human immunodeficiency virus type 1 (HIV)	Tobacco (*Nicotiana tabacum*)	Biolistic method (Stable expression/Chloroplast)	312–363 mg/kg FW	[56]
Japanese encephalitis virus (JEV) envelope protein (E)	Japanese encephalitis virus	Japonica rice (*Nipponbare*)	*Agrobacterium* mediated (Stable expression/Nucleus)	1.1–1.9 μg/mg of total soluble protein	[57]
Hemagglutinin (HA)	Avian influenza (H5N1)	Tobacco (*Nicotiana benthamiana*)	*Agrobacterium* mediated (Transient expression)	0.3 g/kg FW	[58]
Haemagglutinin (HA)	Influenza virus	Tobacco (*Nicotiana benthamiana*)	*Agrobacterium* mediated (Transient expression)	400–1300 mg/kg FW	[59]
Haemagglutinin (HA)	Avian influenza A (H7N7)	Tobacco (*Nicotiana benthamiana*)	*Agrobacterium* mediated (Transient expression)	0.2 g/kg FW	[60]
Structural protein VP2	Infectious bursal disease virus (IBDV)	Tobacco (*Nicotiana benthamiana*)	*Agrobacterium* mediated (Transient expression)	1.0% of total soluble protein	[61]
Structural protein E2	Bovine viral diarrhea virus (BVDV)	Alfalfa (*Medicago sativa* L.)	*Agrobacterium* mediated (Stable expression/Nucleus)	1 μg/g FW	[62]
Bluetongue virus-like particles	Bluetongue virus	Tobacco (*Nicotiana benthamiana*)	*Agrobacterium* mediated (Transient expression)	70 mg/kg FW	[63]
Narita 104 virus virus-like particles	Narita 104 virus	Tobacco (*Nicotiana benthamiana*)	*Agrobacterium* mediated (Transient expression)	0.3 mg/g FW	[64]
Glycoprotein (GP) of PRRSV	Porcine reproductive and respiratory syndrome virus (PRRSV)	*Arabidopsis thaliana*	*Agrobacterium* mediated (Stable expression/Nucleus)	2.74% of total soluble protein	[65]
Matrix protein 2 ectodomain (M2e)	Avian influenza (H5N1)	Duckweed (*Lemna minor*)	*Agrobacterium* mediated (Stable expression/Nucleus)	90–970 mg/kg FW	[66]
Matrix protein 2 ectodomain (M2e)	Avian influenza (H5N1)	Tobacco (*Nicotiana benthamiana*)	*Agrobacterium* mediated (Transient expression)	125–205 mg/kg FW	[67]
Consensus domain III of dengue virus E glycoprotein (cEDIII)	Dengue virus	Tobacco (*Nicotiana benthamiana*)	*Agrobacterium* mediated (Transient expression)	5.2 mg/g FW	[68]
Dengue envelop protein domain III (EDIII)	Dengue virus	Tobacco (*Nicotiana tabacum*)	Biolistic method (Stable expression/Chloroplast)	0.8–1.6% of total soluble protein	[69]
PV3 VLPs	Poliovirus (PV)	Tobacco (*Nicotiana benthamiana*)	*Agrobacterium* mediated (Transient expression)	60 mg/kg FW	[28]
*E. maxima* gametocyte antigen (Gam82)	*Eimeria maxima*	Tobacco (*Nicotiana tabacum*)	*Agrobacterium* mediated (Transient expression)	20 mg/kg FW	[70]
CHIKV E1 and E2	Chikungunya virus	Tobacco (*Nicotiana benthamiana*)	*Agrobacterium* mediated (Transient expression)	8–13 mg/kg of fresh leaf weight	[71]
ZIKV envelope (E) protein	Zika virus (ZIKV)	Tobacco (*Nicotiana benthamiana*)	*Agrobacterium* mediated (Transient expression)	160 μg/g FW	[72]
Porcine circovirus type 2 (PCV-2) capsid protein	Porcine circovirus type 2	Tobacco (*Nicotiana benthamiana*)	*Agrobacterium* mediated (Transient expression)	6.5 mg/kg leaf wet weight	[73]
HIV Env gp140	Human immunodeficiency virus (HIV)	Tobacco (*Nicotiana benthamiana*)	*Agrobacterium* mediated (Transient expression)	5–6 mg/kg FW	[74]
H6 subtype haemagglutinin (HA)	Influenza A virus (H6N2)	Tobacco (*Nicotiana benthamiana*)	*Agrobacterium* mediated (Transient expression)	95 mg/kg FW	[31]
**Antibodies**					
cT84.66	Cancer (tumor marker)	Tobacco (*Nicotiana tabacum*)	*Agrobacterium* mediated (Transient expression)	1 mg/kg FW	[75]
scFvT84.66	Cancer (tumor marker)	Tobacco (*Nicotiana tabacum*)	*Agrobacterium* mediated (Transient expression)	5 mg/kg FW	[75]
scFvT84.66	Cancer (tumor marker)	Rice (*Oryza sativa*)	Biolistic method (Stable expression/Nucleus)	3.8 μg/g FW	[76]
scFvT84.66	Cancer (tumor marker)	Cereal crops (wheat and rice)	Biolistic method (Stable expression/Nucleus)	30 μg/g FW	[77]
BR55-2	Human colorectal cancer	Tobacco (*Nicotiana tabacum*)	*Agrobacterium* mediated (Stable expression/Nucleus)	30 mg/kg FW	[78]
2F5	HIV	Tobacco (*Nicotiana benthamiana*)	*Agrobacterium* mediated (Stable expression/Nucleus)	0.01% of total soluble protein	[79]
2G12	HIV	Tobacco (*Nicotiana benthamiana*)	*Agrobacterium* mediated (Transient expression)	0.3 g/kg FW	[80]
2G12	HIV	Tobacco (*Nicotiana tabacum*)	*Agrobacterium* mediated (Stable expression/Nucleus)	8 mg/L culture medium	[81]
6D8	Ebola virus	Tobacco (*Nicotiana benthamiana*)	*Agrobacterium* mediated (Transient expression)	0.5 mg/g FW	[82]
6D8	Ebola virus	Lettuce (*L. sativa*)	*Agrobacterium* mediated (Transient expression)	0.23–0.27 mg/g FW	[83]
CO17-1AK	Human colorectal cancer	Tobacco (*Nicotiana tabacum*)	*Agrobacterium* mediated (Stable expression/Nucleus)	0.25 mg/kg FW	[84]
Palivizumab-N	Respiratory syncytial virus	Tobacco (*Nicotiana benthamiana*)	*Agrobacterium* mediated (Transient expression)	180 mg/kg FW	[85]
E559	Rabies	Tobacco (*Nicotiana tabacum*)	*Agrobacterium* mediated (Stable expression/Nucleus)	1.8 mg/kg FW (0.04% of total soluble protein)	[86]
pE16	West Nile virus	Tobacco (*Nicotiana benthamiana* ∆XF)	*Agrobacterium* mediated (Transient expression)	0.74 mg/g FW	[87]
pE16scFv-CH	West Nile virus	Tobacco (*Nicotiana benthamiana* ∆XF)	*Agrobacterium* mediated (Transient expression)	0.77 mg/g FW	[87]
E60	Dengue virus	Tobacco (*Nicotiana benthamiana*)	*Agrobacterium* mediated (Transient expression)	120 μg/g FW	[88]
2G12	HIV	Rice (*Oryza sativa*)	Biolistic method (Stable expression/Nucleus)	46.4 μg/g dry seed weight	[89]
8B10 and 5F10	Chikungunya virus	Tobacco (*Nicotiana benthamiana*)	*Agrobacterium* mediated (Transient expression)	20–30 mg/kg FW	[71]
SO57	Rabies virus	Tobacco (*Nicotiana tabacum*)	*Agrobacterium* mediated (Transient expression)	0.014–0.019% of total soluble protein	[90]
cD5	Enterovirus 71	Tobacco (*Nicotiana benthamiana*)	*Agrobacterium* mediated (Transient expression)	50 μg/g FW	[91]
PD1	Cancer	Tobacco (*Nicotiana benthamiana*)	*Agrobacterium* mediated (Transient expression)	140 μg/g FW	[92]
c2A10G6	Zika virus	Tobacco (*Nicotiana benthamiana*)	*Agrobacterium* mediated (Transient expression)	1.47 mg/g FW	[93]
6D8	Ebola	Tobacco (*Nicotiana benthamiana*)	*Agrobacterium* mediated (Transient expression)	1.21 mg/g FW	[93]
HSV8	Herpes simplex virus	Tobacco (*Nicotiana benthamiana*)	*Agrobacterium* mediated (Transient expression)	1.42 mg/g FW	[93]
CHKV mab	Chikungunya virus	Tobacco (*Nicotiana benthamiana*)	*Agrobacterium* mediated (Transient expression)	100 μg/g FW	[94]

**Table 3:** Selected list of various non-pharmaceutical proteins produced in plants.

Recombinant Proteins	Expression System	Transformation Method	Expression Level	Reference
Human serum albumin	Potato (*Solanum tuberosum*)	*Agrobacterium* mediated (Stable expression/Nucleus)	0.25 μg/mg (0.02% of total soluble protein)	[95]
Erythropoietin	Tobacco (*Nicotiana tabacum*)	*Agrobacterium* mediated (Stable expression/Nucleus)	26 pg/mg total protein	[96]
α1-antitrypsin	Rice (*Japonica* rice)	Biolistic method (Stable expression/Nucleus)	4.6–5.7 mg/g dry cell	[97]
Aprotinin	Corn	Biolistic method (Stable expression/Nucleus)	0.069% of total extractable seed protein	[98]
Human-secreted alkaline phosphatase	Tobacco (*Nicotiana tabacum*)	*Agrobacterium* mediated (Stable expression/Nucleus)	1.1 μg/g FW (3% of total soluble protein)	[99]
Collagen	Tobacco (*Nicotiana tabacum*)	*Agrobacterium* mediated (Stable expression/Nucleus)	0.03 g/kg powdered plants	[100]
Human somatotropin (hST)	Tobacco	Biolistic method (Stable expression/Chloroplast)	>7% of total soluble protein	[101]
*Bacillus thuringiensis* (Bt) cry2Aa2	Tobacco	Biolistic method (Stable expression/Chloroplast)	5 mg/g FW (45.3–46.1% of total soluble protein)	[102]
Human serum albumin	Tobacco (*Nicotiana tabacum*)	Biolistic method (Stable expression/Chloroplast)	11.1% of total protein	[103]
Human epidermal growth factor (hEGF)	Tobacco (*Nicotiana tabacum*)	*Agrobacterium* mediated (Stable expression/Nucleus)	34.2 µg/g FW	[104]
Human basic fibroblast growth factor (bFGF)	Soybean (*Glycine max*)	Cotyledonary node explant method (Stable expression/Nucleus)	2.3% of total soluble protein	[105]
Type I interferon (IFNα2b)	Tobacco (*Nicotiana tabacum*)	Biolistic method (Stable expression/Chloroplast)	3 mg/g FW (20% of total soluble protein)	[106]
Human growth hormone (hGH)	Rice (*Oryza sativa*)	Biolistic method (Stable expression/Nucleus)	57 mg/L culture medium	[107]
PlyGBS lysin	Tobacco (*Nicotiana tabacum*)	Biolistic method (Stable expression/Chloroplast)	>70% of the total soluble protein	[108]
Human growth hormone (hGH)	Tobacco BY-2 cells	*Agrobacterium* mediated (Stable expression/Nucleus)	35 mg/L culture medium (2-4% of total soluble protein)	[109]
Human basic fibroblast growth factor (bFGF)	Rice (*Oryza sativa*)	*Agrobacterium* mediated (Stable expression/Nucleus)	185.66 mg/kg	[110]
Lumbrokinase	Sunflower (*Helianthus annuus* L.)	*Agrobacterium* mediated (Stable expression/Nucleus)	5.1 g/kg seeds	[111]
Human acidic fibroblast growth factor 1 (FGF-1)	*Salvia miltiorrhiza*	*Agrobacterium* mediated (Stable expression/Nucleus)	272 ng/g FW	[112]
Glucocerebrosidase (GCase)	Tobacco (*Nicotiana benthamiana*)	*Agrobacterium* mediated (Stable expression/Nucleus)	68 μg/g FW (1.45% of total soluble protein)	[113]
Human acid alpha glucosidase	Tobacco (*Nicotiana tabacum*)	Biolistic method (Stable expression/Chloroplast)	6.38 μg/g FW	[114]
Human basic fibroblast growth factor (bFGF)	Tobacco (*Nicotiana tabacum*)	Biolistic method (Stable expression/Chloroplast)	0.1% of total soluble protein	[115]
Endo-β-1,4-xylanase	Tobacco (*Nicotiana tabacum*)	Biolistic method (Stable expression/Chloroplast)	35.7% of total soluble protein	[116]
β-Glucosidase	Tobacco (*Nicotiana tabacum*)	Biolistic method (Stable expression/Chloroplast)	>75% of total soluble protein	[116]
Osteopontin (OPN)	Tobacco (*Nicotiana benthamiana*)	*Agrobacterium* mediated (Transient expression)	100 ng/g FW	[117]
Dentin matrix protein-1 (DMP1)	Tobacco (*Nicotiana benthamiana*)	*Agrobacterium* mediated (Transient expression)	0.3 µg/g FW	[118]

## 4. Strategies Used for Recombinant Protein Production in Plants

**Figure 1:**
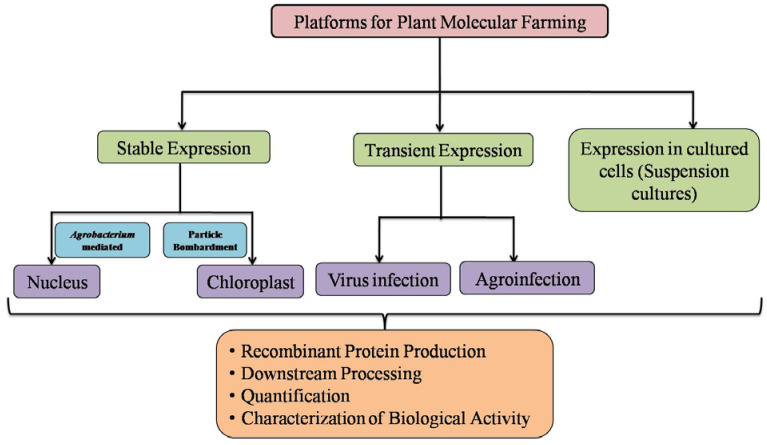
Overview of plant transformation approaches employed for the production of recombinant pharmaceutical and non-pharmaceutical proteins in plants.

The expression methods used for the recombinant protein production in plants can be either stable or transient expression. PMF relies on following approaches for the expression of vaccine candidates, i.e., stable nuclear transformation, stable chloroplast transformation, or transient expression, by using plant viral vectors and stable transformation of hydroponically grown plants in which recombinant proteins are recovered from the medium [119] (Figure 1).

Stable nuclear transformation is the traditional strategy of genetic manipulation in plants for recombinant protein production. The transgene in the plant expression vector can be introduced into the in vitro grown plantlets either with *Agrobacterium tumefaciens*-mediated transformation or particle bombardment, and stable transgenic lines can be developed. The best transgenic line for protein production will be subsequently screened from the pool of transgenic lines. By this method, recombinant proteins can be produced in successive generations, as the transgene has been stably integrated into the plant genome. The model plants such as *Arabidopsis thaliana* and tobacco were more commonly used during the early stages of genetic transformation to develop stable transformants [79]. Stable transformation in plants requires substantial time and is a labor-intensive process, and the protein expression is insufficient to meet the industrial-level protein production. However, the antigen expression in stable transgenic line could be used for developing oral vaccines that could reduce the cost associated with protein purification [120,121].

**Figure 2:**
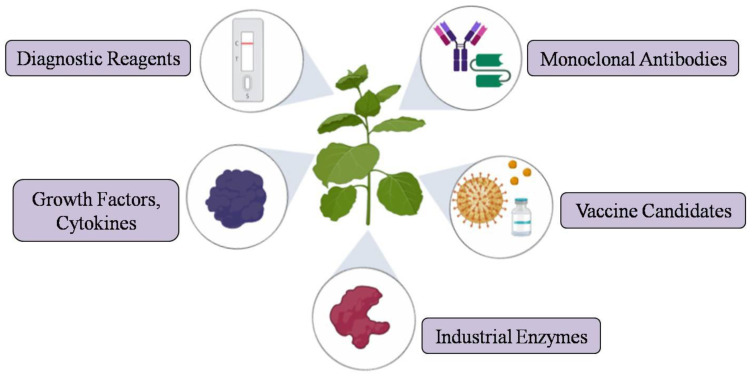
Schematic representation depicting the application of transient expression system for the production of various recombinant proteins.

Alternatively, transient expression based on agroinfiltration or virus-based vectors have been developed to complement transgenic plants that offer rapid and high-level protein expression within a few days. The drawbacks and challenges associated with stable expression, such as insufficient protein expression, time, and consistency, have been overcome by the development of novel strategies involving deconstructed viral vector systems such as MagnICON^®^ technology, geminiviral, and pEAQ, which allows rapid accumulation of recombinant proteins in a short time [12]. Hence, it is considered as a suitable convenient platform, especially for the production of vaccine antigens or monoclonal antibodies against infectious diseases (Figure 2). Gleba et al. (2007) summarized the application of plant viral vectors for the transient expression of heterologous proteins in plants [122]. Plant transient expression holds tremendous potential to produce rapid-response proteins, emergency vaccines, or biologics, which was impressively shown during the Ebola outbreak in 2014. Mapp Biopharmaceutical Inc., USA produced an experimental drug ZMapp, an anti-Ebola antibody cocktail of three chimeric monoclonal antibodies manufactured in tobacco plants (*Nicotiana benthamiana*) to treat humans during the recent Ebola outbreak [123]. During a pandemic situation, in order to cope with a rapidly spreading infectious disease, production methods should meet the demand for production targets of strategic vaccines to control the disease. One of the recent examples is the pandemic, corona virus disease (COVID-19). The virus has spread rapidly, and millions of people have been affected across 6 continents in few months, posing a constant threat to global health. This infection has created a massive demand for diagnostic reagents, vaccines, and therapeutic development. Given the speed advantages, and proven viability of the plant production platform, the transient expression system in particular could be employed to produce recombinant proteins at high levels to meet the sudden demand for production of viral antigens or antiviral proteins that could be used as research reagents, emergency vaccines (SARS-CoV-2 subunit and virus-like particle vaccines), or other biopharmaceuticals to fight against COVID-19 [25,124]. The neutralizing monoclonal antibodies against SARS-CoV-2 could also be produced in plants with minimal investment, which could be used for passive immunotherapy [125]. Recently, Medicago (Quebec, Canada), Kentucky BioProcessing (Owensboro, KT, USA), and iBio (Bryan, TX, USA) joined the global race for developing potential plant-based vaccines for COVID-19 [126]. By using the transient expression platform, recombinant protein production in plants could be scaled up rapidly, and milligram quantities of proteins could be produced in a timeframe of less than 4 weeks after receiving the corresponding gene construct [5,59,127].

Alternately, chloroplast expression focuses on expressing the transgenes in chloroplast by the precise insertion of foreign DNA by homologous recombination into the chloroplast genome. Much progress has been made in chloroplast engineering in recent years. The transformation of the chloroplast genome has many advantages over nuclear transformation which includes higher protein production, lack of gene silencing and position effect, polycistronic mRNA expression, and prevention of transmission of foreign DNA through pollen by uniparental plastid gene inheritance (maternal inheritance) in crop plants [128,129,130,131].

Similar to bacterial and mammalian cells, heterologous protein production can be achieved by using individual suspension of plant cells rather than whole plants. The cell suspension derived from undifferentiated callus grown in liquid medium can be scaled up in bioreactors for large-scale protein production under an aseptic environment. The first USDA-approved poultry vaccine and the first FDA-approved recombinant plant-produced pharmaceutical protein “Elelyso” were produced in tobacco and carrot cell suspension cultures, respectively, which proved the importance and competitiveness of plant suspension culture in high-value protein production in the biopharmaceutical industry [121,132,133,134]. Hairy root cultures are also being explored as an alternative recombinant protein production system due to their ease in protein recovery and low costs. The recombinant proteins are secreted from the transgenic plant roots into the culture medium *viz.,* rhizosecretion; hence, this allows continuous protein production and recovery from the culture medium without the requirement of cell lysis during extraction. Moreover, recombinant proteins produced from root cultures attribute to the improved protein quality and quantity without complex downstream processing that could eventually reduce production costs as well [135]. A recent review on the applications of hairy root cultures for protein production has been extensively discussed by Gutierrez–Valdes et al. (2020) [136].

## 5. Perspectives

Although plants are attractive with several unique advantages, they are unable to compete with the existing microbial and mammalian systems, as both are well established and characterized, especially in terms of GMP manufacturing and regulatory approval in an industrial setting. Even after many years of research, which has shown the proof-of-concept of expressing many therapeutic proteins in plants, the process of producing therapeutic proteins from the lab bench to commercialization is slow. Hence, in order to move forward, the commercial potential and economic sustainability of technology needs to be exploited by developing veterinary vaccines, non-pharmaceutical diagnostic, cosmetic products, and industrial enzymes in plants, as they have a low regulatory burden compared to therapeutic proteins [2,9]. This technology can also be employed to reproduce rapid response vaccines or diagnostic reagents against emerging infections. For the past few years, extensive research has been carried out to combat the several emerging diseases including Zika, chikungunya, Nipah, SARS-CoV, MERS-CoV, and more recently SARS-CoV-2. Even though several efforts have been made for many years to develop effective vaccine candidates for many of those emerging and zoonotic diseases, still, there are no vaccine candidates or therapeutic measures available commercially. Even if a successful vaccine or drug developed against such diseases, it is unlikely that it would have a significant impact on developing and under-developed countries, due to the high cost associated with it, and scalability concern. In such a scenario, a plant-derived vaccine or diagnostic reagent would be a feasible approach to rapidly respond to the demand and need for recombinant proteins. However, harnessing the full potential of this plant molecular farming technology for cost-effective vaccines or drug development will be evident in the upcoming years.

## 6. Conclusions

Plants have both economic and technical advantages over conventional expression systems for the production of pharmaceutical and non-pharmaceutical products. The different PMF technologies such as nuclear, chloroplast expression, viral transfection, and transient expression systems have their unique features, enabling them to address a production of diversified product “targets” with less production constraints in a short time. Many scientific and technical challenges associated with the plant platform were met in recent years. However, the regulatory burden associated with therapeutic protein production is a major barrier that hinders the widespread acceptance of the plant system. Considering the low costs and greater scalability of plant production systems, the commercialization of non-pharmaceutical proteins is straightforward and faster due to lower regulatory challenges. Hence, the universal acceptance of the technology will be strongly influenced by the regulatory framework and restrictions applied to plant-derived products worldwide. The demand for industrially or pharmaceutically useful recombinant proteins, together with demonstrated production capability and economic feasibility of the plant system, suggests a bright future for the plant-made biologics.

## Abbreviations

cGMP	Current good manufacturing practice
COVID-19	Coronavirus disease
FW	Fresh weight
mAb	Monoclonal antibody
MERS-CoV	Middle East respiratory syndrome coronavirus
PMF	Plant molecular farming
PTM	Post-translational modification
SARS-CoV	Severe acute respiratory syndrome coronavirus
SARS-CoV-2	Severe acute respiratory syndrome coronavirus 2
